# Three-Dimensional-Printed Bone Grafts for Simultaneous Bone and Cartilage Regeneration: A Promising Approach to Osteochondral Tissue Engineering

**DOI:** 10.3390/pharmaceutics17040489

**Published:** 2025-04-08

**Authors:** Smiljana Paraš, Božana Petrović, Dijana Mitić, Miloš Lazarević, Marijana Popović Bajić, Marija Živković, Milutin Mićić, Vladimir Biočanin, Slavoljub Živković, Vukoman Jokanović

**Affiliations:** 1 Faculty of Science and Mathematics, University of Banja Luka, 78000 Banja Luka, The Republic of Srpska, Bosnia and Herzegovina; smiljana.paras@pmf.unibl.org; 2 Institute of Nuclear Sciences Vinča—National Institute of the Republic of Serbia, University of Belgrade, Mike Petrovića Alasa 12-14, 11351 Belgrade, Serbia; vjokan@gmail.com; 3 School of Dental Medicine, University of Belgrade, 11000 Belgrade, Serbia; dijana.trisic@stomf.bg.ac.rs (D.M.); milos.lazarevic@stomf.bg.ac.rs (M.L.); dr.marijanapopovic@gmail.com (M.P.B.); marijazivkovic86bg@gmail.com (M.Ž.); slavzivkovic@gmail.com (S.Ž.); 4 Ortoprint doo, 14000 Valjevo, Serbia; milutinmicic@mail.ru; 5 Faculty of Stomatology in Pančevo, University Business Academy in Novi Sad, 21107 Pančevo, Serbia; vladimirbiocanin@gmail.com; 6 ALBOS doo, 11000 Belgrade, Serbia

**Keywords:** nanohydroxyapatite, animal model, bone reconstruction, cartilage regeneration, segmental osteotomy, 3D printing, stem cells

## Abstract

**Background/Objectives**: A novel 3D-printed, bioresorbable bone graft, made of nanohydroxyapatite (nHAP) covered by poly(lactide-co-glycolide) (PLGA), showed strongly expressed osteoinductive properties in our previous investigations. The current study examines its application in the dual regeneration of bone and cartilage by combining with nHAP gel obtained by nHAP enrichment with hydroxyethyl cellulose, sodium hyaluronate, and chondroitin sulfate. **Methods**: In the in vitro part of the study, the mitochondrial activity and osteogenic and chondrogenic differentiation of stem cells derived from apical papilla (SCAPs) in the presence of nHAP gel were investigated. For the in vivo part of the study, three rabbits underwent segmental osteotomies of the lateral condyle of the femur, and defects were filled by 3D-printed grafts customized to the defect geometry. **Results**: In vitro study revealed that nHAP gel displayed significant biocompatibility, substantially increasing mitochondrial activity and facilitating the osteogenic and chondrogenic differentiation of SCAPs. For the in vivo part of the study, after a 12-week healing period, partial resorption of the graft was observed, and lamellar bone tissue with Haversian systems was detected. Histological and stereological evaluations of the implanted grafts indicated successful bone regeneration, marked by the infiltration of new bone and cartilaginous tissue into the graft. The existence of osteocytes and increased vascularization indicated active osteogenesis. The hyaline cartilage near the graft showed numerous new chondrocytes and a significant layer of newly formed cartilage. **Conclusions**: This study demonstrated that tailored 3D-printed bone grafts could efficiently promote the healing of substantial bone defects and the formation of new cartilage without requiring supplementary biological factors, offering a feasible alternative for clinical bone repair applications.

## 1. Introduction

Bone engineering aims to replace damaged bone tissue with materials that seamlessly integrate with the host. A successful bone substitute must provide temporary mechanical support at the defect spot and promote bone regeneration until normal biomechanical function is restored [1]. Substitute material must have the required compressive strength, toughness, and porosity resembling that of natural bone (>50% porosity and >100 mm average pore size) [2]. It also needs to be osteoconductive (to facilitate the supply of nutritional agents through its pores), osteoinductive (to stimulate new bone formation), and osteointegrative (to incorporate into the surrounding bone structures) [3]. Bone substitutes should have a micro-nanometer surface structure crucial for directing cell adhesion and cell proliferation. Bone substitute production aims to imitate the natural bone remodeling processes, so a particular challenge in bone engineering is balancing the resorption rate of a bone substitute with the growth of new bone tissue [2,3,4]. Critically sized bone defects pose a significant challenge in clinical settings, particularly in terms of care and therapy complexity. For this reason, modern medicine has embraced the three-dimensional (3D) printing of bone substitutes with highly precise shapes and sizes tailored to each patient’s specific needs [5].

Nanostructured hydroxyapatite (nHAP) is a widely investigated biomaterial known for its outstanding biocompatibility, and potential to directly stimulate new bone formation, with the absence of toxicity and inflammation [6]. Combining nHAp with natural or synthetic polymers can produce 3D scaffolds for bone grafting, offering sufficient mechanical strength and suitable pore sizes, as well as the ability to stimulate bone growth [6,7,8]. In our previous investigations, we have shown the great potential of nHAP and poly(lactide-co-glycolide) (PLGA)-based bone substitute due to its excellent mechanical properties and ideal solubility, demonstrating complete rabbit calvaria defect healing in 12 weeks [9,10].

Many published papers on bone regeneration [3,11,12] have demonstrated successful large bone defect reconstruction, while a single study offers details on bone tissue reconstruction involving the removal of a full-diameter bone section, including both the proximal and distal parts of the bone through cutting [13]. A significant focus in the development of bone substitutes is to create materials that can promote bone regeneration with practicable, translatable approaches [14]. Bone regeneration in joint areas is especially challenging due to the presence of cartilage whose regeneration demands some modulation of osteoinductive materials. Among the materials showing high potential for cartilage repair, it is well known that hydroxyethyl cellulose improves cell viability, proliferation, and differentiation capacity [15]. Alongside, sodium hyaluronate promotes cell adhesion and proliferation, regulates inflammation, and enhances cartilage regeneration [16], while chondroitin sulfate, as the main component of cartilage extracellular matrix, keeps cartilage healthy by absorbing fluids (particularly water) into the connective tissue. It may also block enzymes that break down cartilage, and it provides the building blocks for the body to produce new cartilage [17].

Driven by the good results that we obtained in our previous study of rabbit’s ulna reconstruction [18], we decided to go a step forward and try to achieve the simultaneous regeneration of bone and cartilage by combining the aforementioned polymers with our nHAP material which already showed good osteoconductive and osteoinductive properties [9,10,18]

Thus, in the current study, we investigated the dual regeneration of bone and cartilage using the gel based on nHAP enriched with hydroxyethyl cellulose, sodium hyaluronate, and chondroitin sulfate (nHAP gel) in combination with the aforementioned ALBO-OS bone substitute [9,10]. The aims of the study were to (1) investigate in vitro biocompatibility and osteogenic and chondrogenic potential of newly synthesized nHAP gel on SCAPs, (2) reconstruct part of the joint in a rabbit model (lateral condyle of the femur), (3) and regenerate combined bone and cartilage defects using a 3D-printed bone substitute made of a novel bioresorbable and osteoinductive nHAP material named ALBO-OS combined with nHAP gel.

## 2. Materials and Methods

### 2.1. Materials Synthesis

Calcium hydroxide (Ca(OH)_2_) powder and ammonium hydrogen phosphate ((NH_4_)_2_HPO_4_) (Merck KGaA, Darmstadt, Germany) were used for the hydrothermal synthesis of nanohydroxyapatite (nHAP). We prepared precursor solutions as a combination of two mixtures: (i) 500 mL of 3.02 cmol aqueous solution of Ca(OH)_2_ (mixture 1) and 500 mL of 2.32 cmol aqueous solution of (NH_4_)_2_HPO_4_ (mixture 2), and added mixture II dropwise into mixture I under simultaneous vigorous mixing on a magnetic stirrer. Finally, we added polyethylene vinyl acetate (PEVA)/polyethylene vinyl versatate (PEVV) (Merck KGaA, Darmstadt, Germany) as a surface-active substance to the obtained common solution. Then, this solution was subjected to hydrothermal treatment at the temperature of 120 °C for 2 h to obtain nHAP particles. Finally, we filtered the solution containing obtained nHAP through a 200 nm pore filter, washed it with deionized water, and dried it at 105 °C for 4 h. The PLGA (50:50, M = 45,000–70,000; Durect Corporation, Cupertino, CA, USA) coating was obtained by pouring a chloroform solution of PLGA over the nHAP particles, which left a thin PLGA film on the particle surface after solvent evaporation. We further used the so-obtained bone substitute, labeled as ALBO-OS, for the fabrication of bone graft by using 3D printing technology according to the corresponding 3D model (this procedure is described in detail in Section 2.2).

After the fabrication of the 3D graft which we have designed for the regeneration of the sub-cartilage bone, a very dense gel, predominantly based on nHAP (94%) (nHAP gel) was synthesized to induce cartilage regeneration. For the production of nHAP gel, we mixed the nHAP powder, obtained as described above, with sodium hexametaphosphate (SHMP) (Merck KGaA, Darmstadt, Germany) as a surface-active substance, during which the SHMP formed a thin coating on the surface of the nHAP particles, and thus preventing their agglomeration. In the next step, hydroxyethyl cellulose (HEC), sodium hyaluronate (SH), and chondroitin sulfate (CH) (all purchased from Merck KGaA, Darmstadt, Germany) were added to so processed nHAP. Ratios of the components (in mass %) were SHMP:HEC:SH:CH:nHAP = 1:2:1.5:1.5:94. After that, we processed the given mixture in a planetary mill with ceramic balls using ceramic lining. The ratio between the gel mixture and balls with a diameter of 1 cm was 1:10. A rotational speed of 250 rpm resulted in an acceleration of about 6.5 g (d is gravitation acceleration), while milling time was 39 min. After milling, we added a small amount of water and glycerin dropwise to obtain a very dense complex gel, which will be applied onto the porous surface of the previously printed construct.

### 2.2. Three-Dimensional (3D) Modeling and Printing of Bone and Cartilage Grafts

#### 2.2.1. CBCT Data to Printable 3D Model Transformation

A preoperative cone beam computed tomography (CBCT) scan of the rabbit’s ulna was obtained via the Scanora 3D scanner (Soredex, Tuusula, Finland), while the animal was under anesthesia (as described in Section 2.4) using the following parameters: 13 mA, 90 kV, and a voxel resolution of 0.2 mm. After scanning, we generated a Standard Tessellation Language (STL) file of target bones and cartilage tissue from the CT data using the Slicer software version 4.3.1 and the Blender software, version 4.3. (Autodesc Inc., San Francisco, CA, USA). Thus, the obtained STL files were imported into Autodesk 3D Max 2010 (Autodesc Inc., San Francisco, CA, USA).

**Figure 1:**
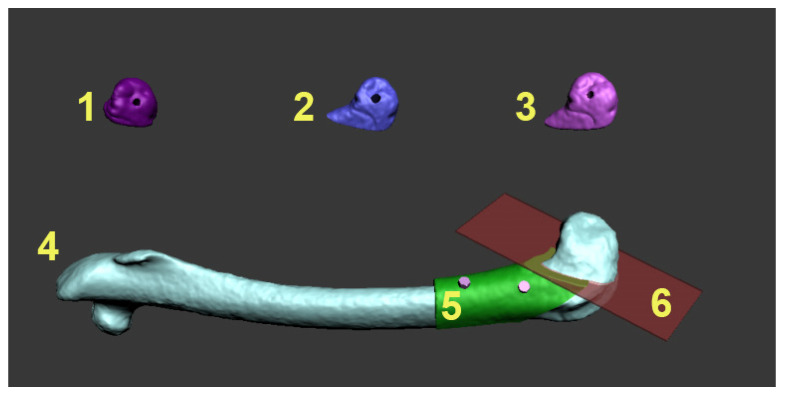
Surgery planning procedure in the Autodesk 3D Max 2010 software. Numbers 1–3: The identification numbers of every experimental animal and 3D model of the lateral femur reconstruction graft with the fixation hole. 4: The 3D appearance of the rabbit’s femur. 5: The surgical guide for the resection of the lateral condyle (green) with the fixation holes for fixation screws (pink). 6: The planned resection plane (transparent red) of the lateral condyle.

To prepare the grafts for the experiment, we planned a diagonal resection on the distal end of the rabbit’s femur to dissect the lateral condyle, as visualized in Figure 1. Based on the surface 3D model of the bone, a graft was modeled layer by layer using a maze-forming algorithm.

#### 2.2.2. Modeling and 3D Printing of the Constructs with the Desired Porosity

**Figure 2:**
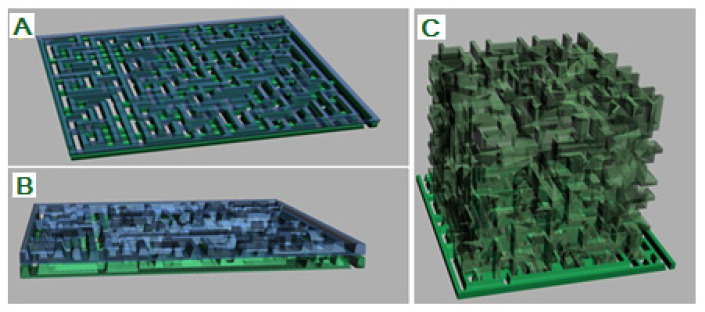
Three-dimensional modeling of the desired porosity: (**A**)—Forming the planar maze structure in X- and Y-axis directions in software and stacking it in Z direction (green and blue maze layers); (**B**)—Extruding maze structure in Z-axis direction to form a 3D structure and stacking the formed structures on each other in Z-axis direction using Autodesk 3D Max (green and blue maze layers); (**C**)—After removing the outer walls of the stacked maze structure in Autodesk 3D Max, the 3D model of the bone construct structure with 50% porosity and trabecular thickness of 225 μm is given in transparent form.

To achieve the desired porosity and pore interconnectivity in the custom bone and cartilage replacement constructs, we applied the randomized Kruskal’s maze-generating algorithm (RKA) using Python software, version 3.12.0. (Figure 2A). The RKA generated a 15 × 15 mm planar maze in the x- and z- axes, allowing control over porosity and “bone” volume by adjusting the ratio of maze wall thickness to path width. The walls were set at 225 μm to ensure pore interconnectivity by removing enclosed cells.

Then, the 2D maze structure was extruded by 225 μm along the Z-axis, creating a 3D object. This 3D maze generation was repeated and stacked along the Z-axis until it reached the bone construct’s dimensions (X—15 mm, Y—15 mm, and Z—25 mm), providing random vertical interconnectivity. This produced a 3D bone construct with 50% porosity and trabecular thickness of 225 μm, where maze walls formed trabeculae and paths represented voids. In this way, we prepared 3D models that formed an inner structure of 3D grafts, with a planned trabecular thickness of 225 μm which was expected to have a 27% standard deviation after 3D printing, according to the methodology established by Micic et al. [18] (Figure 2).

The 3D maze and bone and cartilage construct surface 3D models were imported into Autodesk 3D Max, where we merged them with a Boolean union operation so that cartilage surface models gave outer margins or shape to the resulting cartilage 3D model and maze 3D model gave an inner structure. G-code was prepared for 3D printing in the Slice3R software with settings of 0.2 mm layer height, 100% infill, 10 mm/s print speed, zero shell thickness, and no support material. Three-dimensional printing was performed as previously described by Micic et al. [18] using the 3D printer described in Appendix A and the Pronterface software, version 2.0.1, with a paste made of ALBO-OS particles less than 300 µm and PLGA as a support material, using 0.3 mm nozzle diameter. The process of printing the construct was set so that the concentration of ALBO-OS particles gradually increases from 20 to 80% from the construct periphery towards the center, with the maximum concentration of PLGA on the construct surface. The three-dimensional printing technique used has been verified by micro-CT characterization of the 3D printed constructs.

For the rabbit’s femoral condyle graft, we rubbed the prefabricated nHAP gel (described in Section 2.1) onto the porous surface of the printed construct. Following the printing of 3D bone grafts with the precisely matched shape of the desired defect, the bone models underwent UV sterilization for 12 h.

### 2.3. In Vitro Study on SCAPs

#### 2.3.1. Mitochondrial Activity

In vitro experiments on stem cells were approved by the Ethics Committee of the School of Dental Medicine, University of Belgrade (number: 36/8). Mitochondrial activity assay was performed following ISO standard 10993-5 [19]. We prepared experimental samples as gel emulsions in growth medium (1%, 0.5%, 0.25%, 0.125%).

Stem cells of the apical papilla (SCAPs) were isolated and characterized as previously described [18]. Briefly, we excised the apical papilla from the tooth apex, after which we fragmented tissue into 1 mm³ pieces and placed it in Dulbecco’s modified Eagle medium supplemented with 10% fetal bovine serum and 1% antibiotic–antimycotic solution (all from Thermo Fisher Scientific, Waltham, MA, USA). The cells were maintained at 37 °C in a humidified atmosphere with 5% CO_2_. The culture media was changed every two to three days. Upon achieving 80% confluence, the cell cultures were passaged. We utilized the fifth-passage cells for the investigations.

SCAPs were seeded in 96-well plates, 5000 cells/well. The next day we added serial solutions of nHAP gel material in the wells, using cells cultured in a growth medium as a control. The plates were incubated at 37 °C and 5% CO_2_ for 24 h after which the mitochondrial activity of the materials was evaluated through the (3-([4,5-dimethylthiazol-2-yl]-2,5-diphenyl-tetetrazolium bromide (MTT) cell metabolic activity assay (Sigma-Aldrich, St. Louis, MO, USA). To quantify cell metabolic activity, we removed the growth medium, and added a freshly prepared growth medium containing 5 mg/mL MTT. The plates with cells were incubated in the dark at 37 °C for 4 h, after which we removed the medium and added 100 μL of dimethyl sulfoxide (DMSO) (Sigma-Aldrich, St. Louis, MO, USA) to the wells. The absorbance was measured at 550 nm in a microplate reader (RT-2100c, Rayto, Schenzhen, China).

#### 2.3.2. Osteogenic and Chondrogenic Differentiation

SCAPs were seeded in 96-well plates in a concentration of 10,000 cells/well, in a growth medium. The next day, as the cells reached 80% confluence, the growth medium was discarded, and the materials’ solution in concentration 0.125% was added to the corresponding wells. Materials were dissolved in osteogenic (StemMACS™ OsteoDiff Medium, Miltenyi Biotec, Bergisch Gladbach, Germany) or chondrogenic medium (StemMACS™ ChondroDiff Medium, Miltenyi Biotec, Bergisch Gladbach, Germany). As a control, cells cultured only in osteogenic or chondrogenic medium were used. The plates were incubated at 37 °C and 5% CO_2_ for 7 days, after which the cells were fixed with 4% neutral formalin buffer for 15 min., and stained with 2% Alizarin-Red Staining solution (ARS, Sigma-Aldrich, St. Louis, MO, USA), or with 1% in 3% acetic acid Alcian Blue solution (Sigma-Aldrich, St. Louis, MO, USA). ARS and Alcian Blue bounds to cultures were extracted by incubation with 250 μL of 1% hydrochloric acid in 70% ethanol for 20 min. The absorbance was measured at 405 nm in a microplate reader (RT-2100c, Rayto, Schenzhen, China).

### 2.4. In Vivo Customized Bone Model Implantation in Rabbit Joint

**Figure 3:**
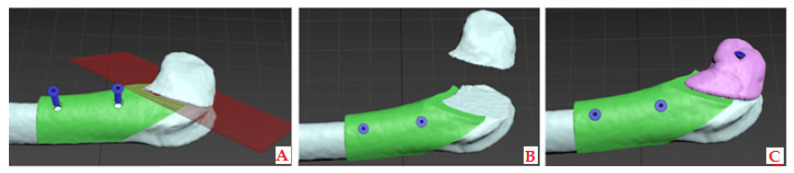
Virtual planning of the surgery procedure: (**A**)—surgical guide (SG) (green) placement on the rabbit femur (white), cutting plane (transparent red) predicted by the position of the SG, fixation screws (blue) for the SG; (**B**)—SG (green) fixed on the femur (white), ensuring the precise cut, lateral condyle of the femur is removed; (**C**)—SG (green) still in place of the planned defect site, 3D graft (pale pink) is placed and fixed with the fixation screw (blue).

The experiment included three adult New Zealand white rabbits, 4-month-old, weighing approximately 3.2–4.1 kg, following the EU guidelines (86/609/EEC) regarding the use of laboratory animals in line with the principles of good laboratory practice and the principles of care and use of laboratory animals (NIH publication 85–23, revised in 1985). The experimental research was approved by the Ethics Committee of the Faculty of Veterinary Medicine, University of Belgrade (number: 323-07-06340/2019-05/1). The first step involved intramuscular premedication (combination of Ketamidor (ketamine hydrochloride) 10%, Richter Pharma Ag, Austria, 35 mg/kg, and xylazine (Xylased) 2%, Bioveta, Czech Republic, 5 mg/kg) and CBCT scanning of all three laboratory animals (left ulna) to obtain images for designing customized bone models. Butorphanol (Richter Pharma Ag, Wels, Austria) was administered intramuscularly at a dose of 0.1 mg/kg for analgesia. Under local anesthesia (Lidocaine-chloride 2%, Galenika a.d., Belgrade, Serbia), a linear skin incision of 5.5 cm was made in the left joint region of all the rabbits. The joint was revealed by gently lifting the overlying soft tissues. After removing a predetermined section of lateral femur condyle, 3D bone grafts were implanted into the defect site without using any stem cells or growth factors. Namely, the defect was created at a predetermined distance of 1 cm from the top of the lateral femur condyle to ensure accurate placement of the 3D-printed graft. A segmental osteotomy of the lateral femur condyle was performed in all three rabbits. The 3D printed graft with applied nHAP gel on its surface was placed at the site of the osteotomy defect and fixed with a titanium screw (KLS Martin, Jacksonville, FL, USA), 12 mm long and 1 mm in diameter, through the graft hole that was previously precisely designed. A virtual planning of the surgical procedure is shown in Figure 3.

Following the placement of the 3D bone graft, mediolateral X-rays were acquired using a ZooMax White DR system, version 1.0 (Control-X Medical, Ltd., Dunakezsi, Hungary) to verify the accuracy of its positioning. The imaging parameters were 47 kV, 6.4 mAs, and 70 cm focal-film distance. X-ray images were digitized using a CR 10-X (Agfa HealthCare NV, Mortsel, Belgium). The soft structures were repositioned, and the surgical wound was closed with simple sutures (Vicryl, Ethicon, 3-0). Postoperatively, the rabbits were kept in cages with unrestricted access to food and water. Air temperature in the vivarium was kept at 23 ± 3 °C, with a humidity of 55 ± 5%, and a 12/12 light–dark cycle. The rabbits showed no signs of discomfort or loss of appetite five days after surgery. The incision site and the surrounding skin were clean. The skin sutures were removed ten days postoperatively. Five consecutive days after the intervention, the rabbits received buprenorphine (Alkaloid AD, Skopje, Republic of North Macedonia) 0.1 mg/kg (twice a day) and oxytetracycline (Dopharma B.V., Zalmweng, The Netherlands) 20 mg/kg subcutaneously to control pain and infection. The rabbit grimace scale, a standardized behavioral coding system that shows facial expressions, was used to assess pain. The rabbits were sacrificed 12 weeks following the experimental procedure when both joints were explanted from all the rabbits. The right joint served as a control, while the left joint with the implanted 3D bone graft was examined as the experimental one. The sampled material was fixed in 4% neutral buffered formalin.

#### 2.4.1. Histological and Histomorphometric Analysis

The bone tissue samples were prepared for histological analysis using standard light microscopy techniques, including fixation in 4% buffered formaldehyde, decalcification in formic acid, dehydration, and paraffin embedding. Longitudinal sections, 4 μm thick, were stained with hematoxylin and eosin. Immunohistochemical staining for osteocalcin and collagen (Picrosirius red) was also performed. Given the potential differences in regeneration efficiency between the cartilage and bone, all the histological and histomorphometric parameters were evaluated in both tissues. Morphometric evaluation was performed using a final magnification of 400×. Two-dimensional photographs were taken using a digital camera Leica DFC295 (Leica Microsystems, Wetzlar, Germany). Histological analysis was performed using light microscopy (Leitz Laborlux S fluorescence microscope, Ernst Leitz GMBH, Wetzlar, Germany). Histomorphometric analysis was performed using the software package Leica University Suite, version 4.3 (Leica Microsystems, Wetzlar, Germany).

#### 2.4.2. qPCR Analysis of Tissue Samples

##### RNA Isolation and Reverse Transcription

The tissue samples (bone and cartilage) were minced (1 cm^3^ pieces), and total RNA was extracted by using TRIzol Reagent (Invitrogen, Thermo Fisher Scientific, Waltham, MA, USA) according to the manufacturer’s recommendations. The RNA concentration was measured using the microvolume spectrophotometer (BioSpec–nano Microvolume UV–Vis Spectrophotometer; Shimadzu Scientific Instruments, Columbia, MD, USA). Oligo d(T) primer and the RevertAid First Strand cDNA Synthesis Kit (Thermo Fisher Scientific, Waltham, MA, USA) were used to synthesize cDNA from 2 µg of total RNA.

##### Gene Expression Analysis of Osteogenic and Chondrogenic Differentiation

Real-time quantitative polymerase chain reaction (qPCR) was performed using the first strand cDNA, 0.2 μM forward and reverse primers, and the SensiFAST SYBR Hi–ROX Kit (Bioline, London, UK). The expression of the following markers was analyzed: bone morphogenic protein 4 (*BMP4*), runt-related transcription factor 2 (RUNX2), and alkaline phosphatase (*ALP*) as osteogenic markers; and integral membrane protein 2A (*ITM2A*), forkhead box C1 (*FOXC1*), and SRY-Box Transcription Factor 9 (*SOX9*) as chondrogenic markers. The housekeeping gene glyceraldehyde-3-phosphate dehydrogenase (*GAPDH*) was used as a reference. The relative gene expression values were calculated using the 2^−ΔCt^ method [20]. The sequences of all the primers used in this study are given in Table 1.

### 2.5. Statistical Analysis

The software package GraphPad Prism version 9 was used for the analyses (GraphPad Software, Inc., Boston, MA, USA). A non-parametric Mann–Whitney U-test was used in the study, given the small sample size. The results are presented as median with interquartile range (IQR). * *p* < 0.05, ** *p* < 0.01, *** *p* < 0.001, and **** *p* < 0.0001 denote statistical significance.

## 3. Results

### 3.1. In Vitro Results on SCAP Cells

#### 3.1.1. Mitochondrial Activity

**Figure 4:**
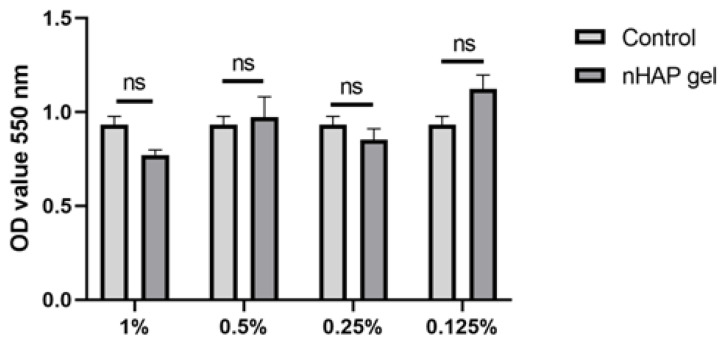
Mitochondrial activity of grade concentrations of nHAP gel and control, after 24 h, ns—not significant.

After 24 h, all the concentrations stimulated the cells to have mitochondrial activity over 70%, which is considered to be a safety limit for materials in vitro [19]. There were no statistically significant differences between the nHAP gel and control groups (Figure 4).

#### 3.1.2. Osteogenic and Chondrogenic Differentiation

**Figure 5:**
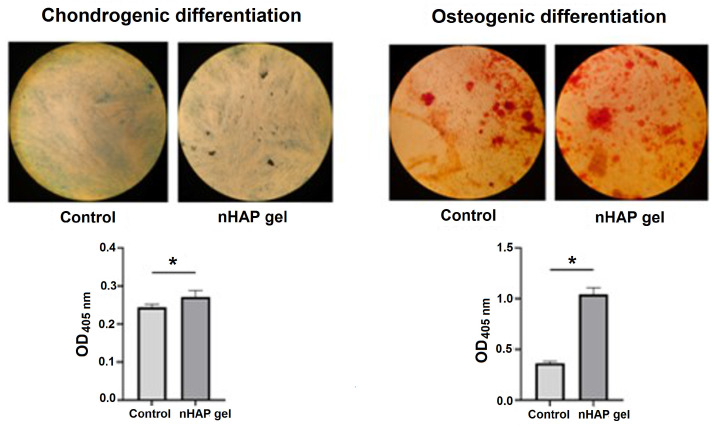
Chondrogenic and osteogenic differentiation of SCAPs after 7 days of culturing in induction mediums, enriched with 0.125% of nHAP gel, and control. Magnification 40×; * *p* < 0.05.

Since the concentration of 0.125% stimulated cell activity, it was chosen for further experiments. Cells were cultured in induction mediums for 7 days to observe the potential of materials to accelerate differentiation in the early stages of culturing. After 7 days of culturing cells in an osteogenic medium, the group treated with nHAP gel significantly stimulated osteogenic differentiation, *p* < 0.001, in comparison to the control. Mineralized globules were more prevalent and evenly distributed on the surface of the cells treated with nHAP gel. Also, nHAP gel significantly stimulated the cells to chondrogenic differentiation, *p* < 0.05, in comparison to the control (Figure 5).

### 3.2. Stereological and Histological Findings

The implanted material proved to be very soluble for fixatives and brittle for cutting on a microtome, in the standard cytohistological procedure for analysis by light microscopy, which primarily influenced its detection. The material appeared torn and crumpled in histological sections, often only in fragments or pieces that remained closest to bone and cartilage cells (marked as “defect area” on micrographs).

**Figure 6:**
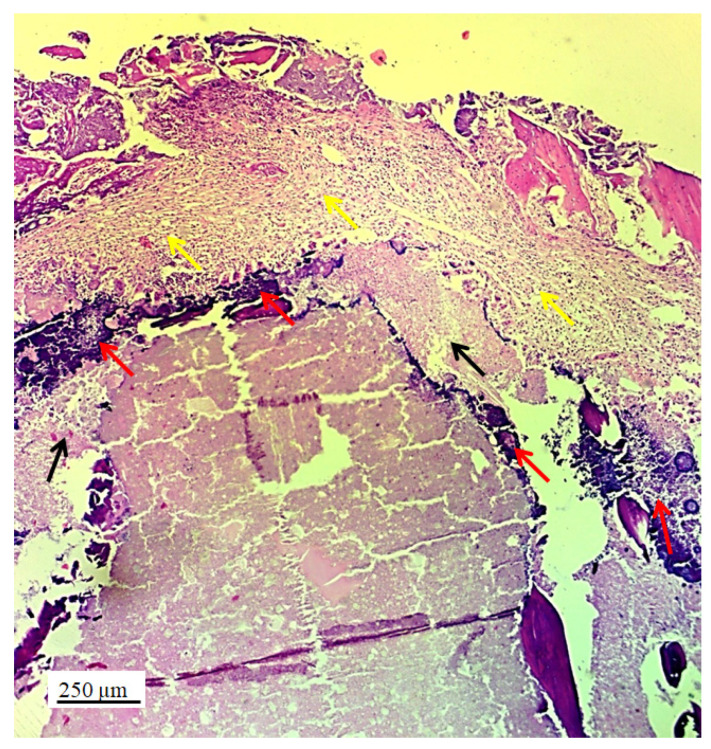
Longitudinal section of lateral femur condyle with implanted 3D graft, H&E, magnification 20×; locations of graft (red arrows), chondrocytes (black arrows), and fibroblasts (yellow arrows).

**Figure 7:**
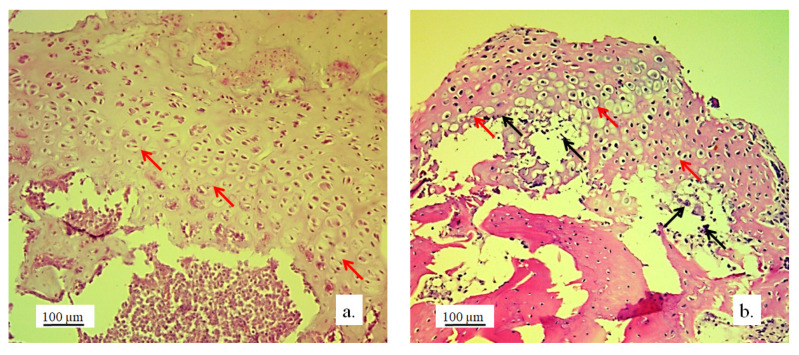
Cartilaginous tissue in longitudinal sections of the lateral femur condyles: (**a**) control and (**b**) with 3D graft, H&E, magnification 50×; chondrocytes (red arrows) and lacunae with fragments of implanted material (black arrows).

**Figure 8:**
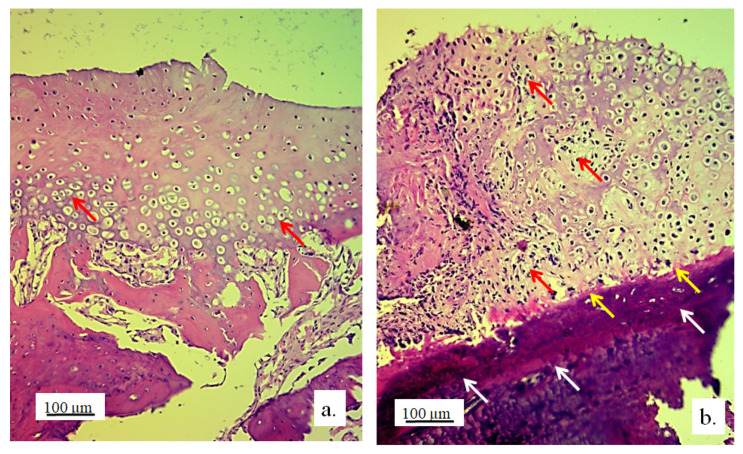
Cartilaginous tissue in the longitudinal section of the lateral femur condyle: (**a**) control and (**b**) with 3D graft, stained with immunohistochemical technique Picrosirius red, magnification 50×; chondrocytes (red arrows), fibroblasts (yellow arrows), and fragments of graft (black arrows).

**Figure 9:**
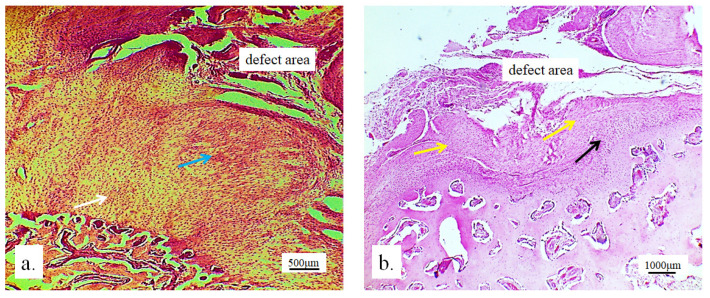
(**a**) Longitudinal section of the articular femur surface with 3D graft: newly formed irregular cartilage (blue arrow) and regular cartilage in contact with bone tissue (white arrow), immunohistochemical Goldner stain, magnification 10×; (**b**) longitudinal section of the articular femur surface with 3D graft: regular cartilage structure (black arrow) and newly formed irregular cartilage structure (yellow arrows), Toluidine blue stain, magnification 10×.

**Figure 10:**
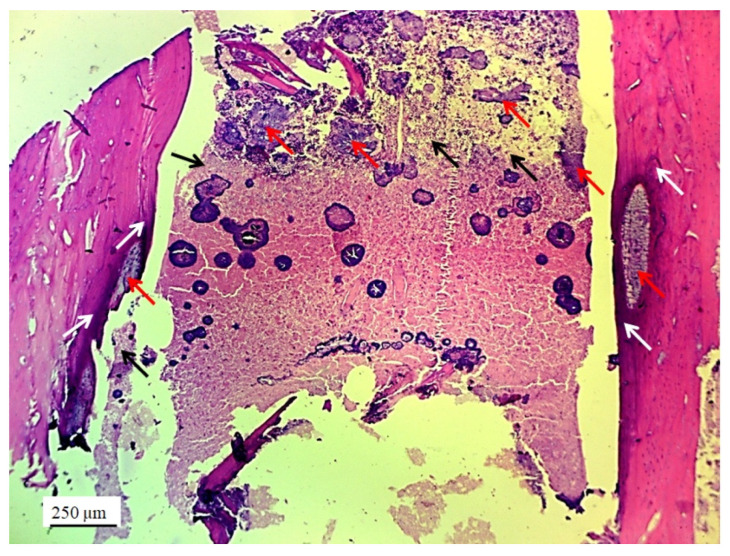
Longitudinal section of a femur with 3D graft, stained with immunohistochemical technique Picrosirius red, magnification 20×: osteocytes (white arrows), fibroblasts (black arrows), and location of implanted material (red arrows).

**Figure 11:**
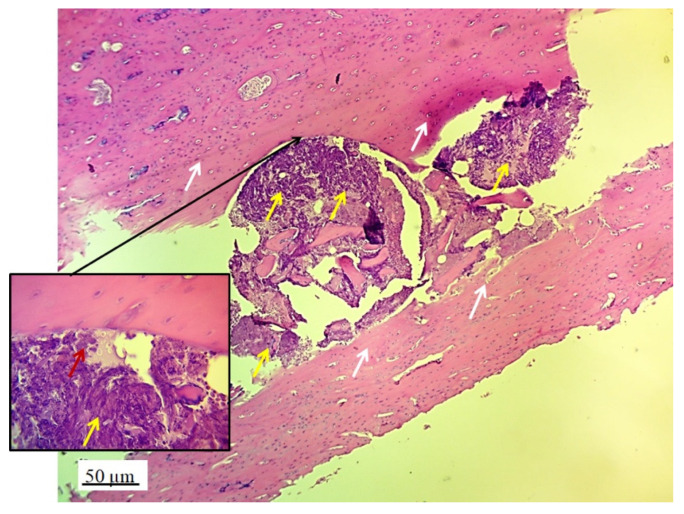
Cross-section of femur with implanted 3D graft, H&E, magnification 100×: osteocytes (white arrows), fibroblasts with cytoplasmic extensions (red arrow), magnified junction of femur bone tissue and 3D graft (black arrow and black square), and location of implanted graft (yellow arrows).

**Figure 12:**
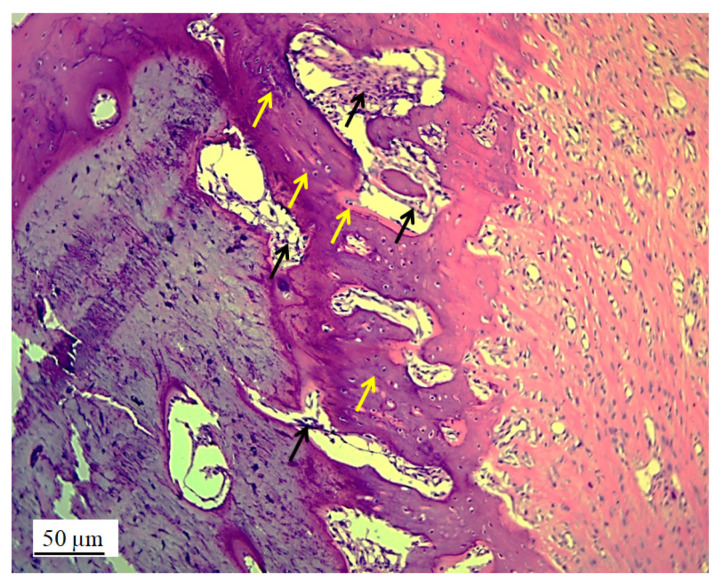
Cross-section of lateral femur condyle with implanted graft, stained with immunohistochemical technique Picrosirius red, magnification 100×: osteocytes (yellow arrows) and location of implanted material (black arrows).

**Figure 13:**
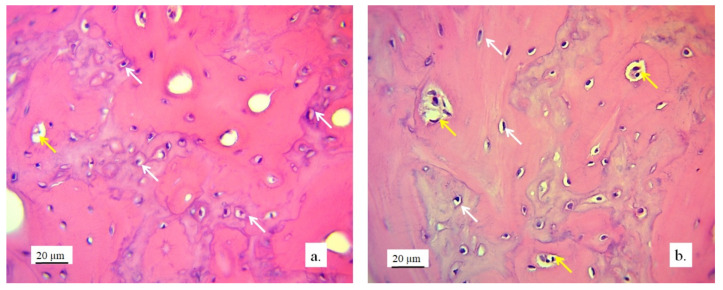
Cross-section of lateral femur condyle in control (**a**) and with implanted graft (**b**), H&E, magnification 200×: osteocytes (white arrows) and blood vessels (yellow arrows).

**Figure 14:**
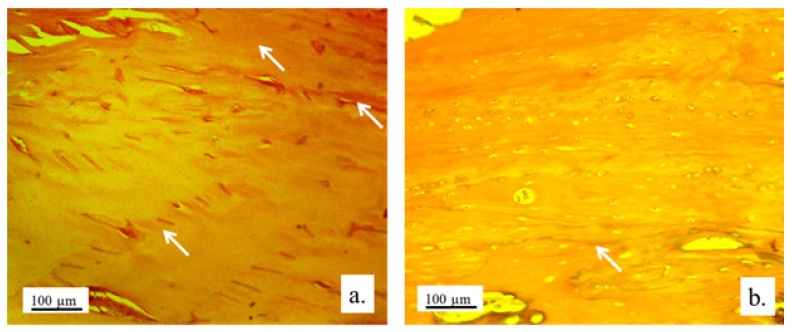
Immunoreactivity to osteocalcin. Representative micrographs of femur tissue stained with an immunohistochemical technique for the visualization of osteocalcin: longitudinal section of femur without an implant (**a**) and femur with an implanted graft (**b**). White arrows indicate the sites of immunoreaction of bone tissue to osteocalcin, magnification 100×.

**Figure 15:**
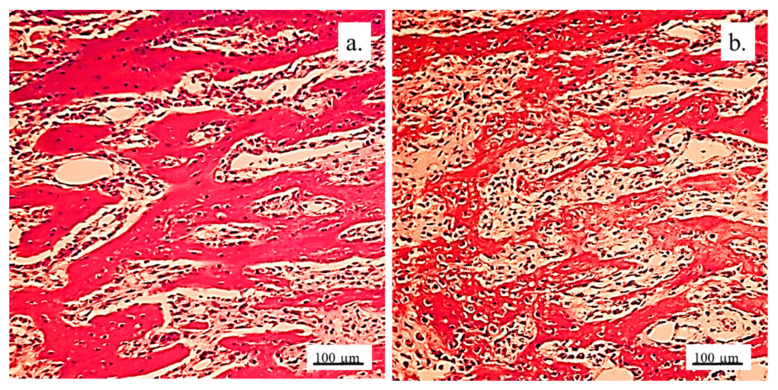
Collagen deposition in bone tissue. Representative micrographs of bone tissue stained with histochemical technique Picrosirius red: control bone (**a**) and bone with implanted graft (**b**), 100× magnification.

However, in the micrographs, a few remaining parts of the graft nanoparticles were observed, as well as the creation of new cartilage and bone tissue. The stereological (Table 2) and histological (Figure 6, Figure 7, Figure 8, Figure 9, Figure 10, Figure 11, Figure 12, Figure 13, Figure 14 and Figure 15) analyses of the longitudinal and transverse sections of the rabbits’ joints revealed the presence of newly formed bone tissue and cartilage.

#### 3.2.1. Hyaline Cartilage in the Rabbit Joint Region

Hyaline cartilage in the region of the implanted joint was shown to be regenerated 12 weeks after the implantation of the 3D-printed graft. The stereological (Table 2) and histological (Figure 6, Figure 7, Figure 8, Figure 9 and Figure 10) analysis of longitudinal and transverse sections of the rabbit joint showed the presence of newly formed cartilage.

Numerous new chondrocytes and a significant layer of newly formed cartilage were observed in the hyaline cartilage of the lateral femur condyle. The presence of new chondrocytes strongly indicated the active reparation and advanced regeneration of the defect replaced with the new 3D graft (Table 2). A large number of fibroblasts and reticulocytes were observed—the cells active in tissue repair as mediators of wound healing (Figure 6).

The architecture of the hyaline cartilage on the surface of the lateral femur condyle in the control group (without the 3D graft) showed more chondrocytes of larger dimensions (Figure 7a) compared to the chondrocytes of the experimental group with the implanted 3D graft. Chondrocytes of smaller dimensions, fewer in number, altered, irregular, flattened, spindle-shaped, and balloon-shaped with wide lacunae (Figure 7b) were observed in the experimental groups. The implanted material still remained in the form of small clusters around the edges of lacunae (Figure 7, black arrows).

Chondrocytes in the cartilaginous tissues of the lateral femur condyle that was replaced with the 3D graft did not have a regular linear and narrow arrangement as in the control group (Figure 8a). They were scattered, irregular, and disordered (Figure 8b). The newly formed layer of cartilage which covered the femur defect was not thinner than in the control group (Figure 8). The NCR of chondrocytes with the 3D graft was high (Table 2), which all together indicates the presence of a process of cartilage tissue regeneration and chondrogenesis near the graft.

The articular surface of the rabbits’ femur with the 3D graft (Figure 9, defect area) was filled with newly formed irregular reticular cells and fibroblasts in cartilage tissue (Figure 9a, blue arrow). Immature chondrocytes and fibroblasts penetrated the pores of the 3D graft on the articular surfaces of the femur and thus proved the presence of chondrogenesis, the process of cartilage regeneration. The white arrow (Figure 9a) shows regular cartilaginous tissue that is covered by newly formed cartilage on the upper side, while contact with bone tissue is visible on the lower side.

The longitudinal section of the joint with the 3D graft showed the penetration of fibroblasts through the pores of the implanted graft (Figure 9b). In the same image, young reticulocytes (yellow arrows) and chondrocytes (black arrows) were detected at the site of new cartilage formation and thus prove the presence of chondrogenesis.

#### 3.2.2. Bone Tissue in the Rabbit Joint Defect Region (Sub-Cartilage Region)

During a 12-week healing period, there were no signs of necrosis, infection, or bleeding. The bone tissue showed a good degree of regeneration and reparation of the defect filled with the 3D graft (Table 2). New osteocytes were numerous even though the young bone layer was thin.

The presence of cysts, necrosis, infiltration of blood cells, or immune response was not observed in the bone defect replaced by the 3D graft. Figure 10 and Figure 11 represent the longitudinal and cross-sectional micrographs of a femoral defect filled with the 3D graft. After 12 weeks, the connection between the damaged bone tissue and the implanted 3D graft was visible. No lymphocytes or cysts at the contact of bone and implanted material were observed (Figure 11). Only fibroblasts with long cytoplasmic extensions that bind material to the bone were detected. Young osteocytes and fibroblasts were observed as mediators of osteogenesis.

Fragments of the implanted material were detected on the cross-sections of the lateral femoral condyle with the implanted 3D graft. The presence of dark blue-colored young osteocytes was also observed (Figure 12).

The architecture of the bone tissue with implanted graft was changed, the osteocytes were fewer and had different shapes compared to the osteocytes in the control group (Figure 13). NCR of osteocytes in bone with the implanted grafts was elevated (Table 2). This fact, as well as the presence of fibrocytes, indicated the acceptance processes of the implanted material by the bone tissue. Blood vessels in the bone tissue with the implanted graft had a higher volume density and diameter compared to the control group. (Figure 13). Signs of angiogenesis in the bone tissue near the implanted graft indicated reparation and regeneration processes.

The detection and visualization of osteocytes was performed by the immunohistochemical staining of osteocalcin. Figure 14a shows a longitudinal section of femur tissue without an implant, where excellent immunoreactivity to osteocalcin was observed compared to weaker reactivity to osteocalcin in Figure 14b, where the graft was implanted. In Figure 14b, the presence of osteocalcin was observed, which proved that the process of osteogenesis was present in the newly formed bone with implants.

The histochemical analysis revealed a small amount of mineralized bone tissue and a larger quantity of unmineralized bone, indicating ongoing osteogenesis. Additionally, a significant portion of the newly formed bone exhibited lamellar organization with vital osteocytes arranged concentrically around the Haversian canals. The presence of immature bone pointed to the active remodeling of bone tissue. The newly formed bone predominantly displayed trabecular architecture, with new bone marrow occupying the spaces between the trabeculae.

Visualization of collagen was performed by immunohistochemical staining with Picrosirius red. Figure 15 shows a very strong immunoreactivity to collagen in the control group, as well as in the experimental group. This indicated that osteogenesis was present in the newly formed bone with implanted graft since collagen was present in the extracellular matrix of the newly formed bone tissue.

### 3.3. qPCR Analysis of Osteogenic and Chondrogenic Markers

**Figure 16:**
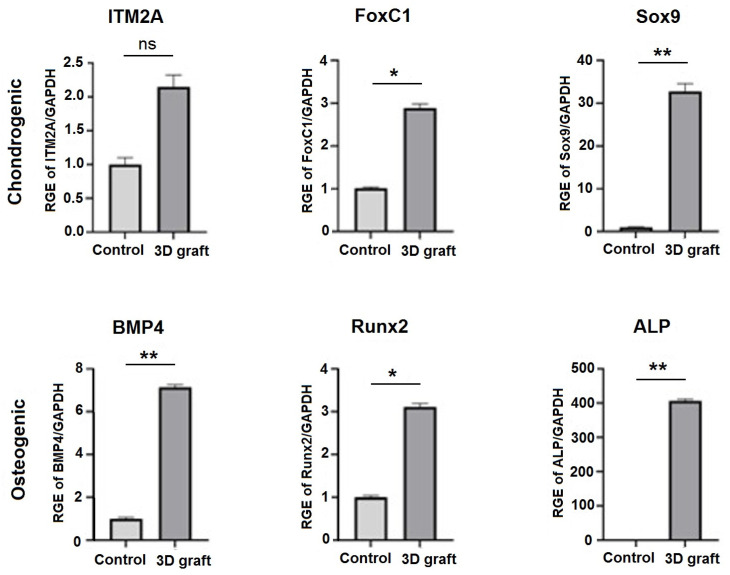
Relative gene expression (RGE) levels of chondrogenic (*ITM2A*, *FOXC1*, and *SOX9*) and osteogenic (*BMP4*, *RUNX2*, and *ALP*) markers in tissues with implanted material compared to control; ns—not significant, * *p* < 0.05, and ** *p* < 0.01.

Markers of osteogenic differentiation (*BMP4*, *RUNX2*, and *ALP*) were upregulated in the presence of material compared to control (Figure 14). The expression levels of BMP4 and Runx2 were significantly higher in comparison to control (*p* < 0.05). In the presence of 3D construct, markers of chondrogenic differentiation (*ITM2A*, *FOXC1*, and *SOX9*) were upregulated, especially *FOXC1* and *SOX9* (*p* < 0.0001) (Figure 16).

## 4. Discussion

Our results demonstrated that the 3D-printed bone graft acts as a scaffold that imitates bone tissue structure and facilitates the migration, attachment, and reproduction of bone cells and the formation of new bone on the one hand, as well as the regeneration of cartilage on the other. The introduction of complex nHAP gel to established nHAP-PLGA construct enabled the achieving of simultaneous regeneration of cartilage and bone, thus providing a complex reconstruction of femur condyle.

After 12 weeks of healing, mature mineralized lamellar bone with Haversian osteons and blood vessels, as well as mature bone marrow, were observed at the periphery of the defect, indicating that almost complete peripheral bone defect regeneration was achieved. These results are consistent with those of Tang et al. [21], which described almost complete bone regeneration using a new material containing bioactive glass with a growth factor recombinant human BMP-2. The newly formed bone tissue strongly suggests that active osteogenesis and remodeling were ongoing 12 weeks post-implantation. Runx2, as a master osteogenic transcription factor that regulates the BMP4 pathway [22] was upregulated as well as BMP4 in tissue samples where the construct was implanted. The upregulation of osteogenic markers corresponds to osteoblast differentiation and active bone formation in the presence of 3D constructs in the tissue. Considering the inflammatory cell count as the most important parameter of bone substitute biocompatibility [13], the results indicate the significant biocompatibility of the 3D bone graft and complete bone regeneration.

The optimal degradation rate of the 3D bone graft, which corresponds to the new bone formation rate, is crucial for the osteoinductive potential of the bone. Also, many authors have made efforts to reconstruct large bone defects [11,12,23,24], which can be challenging compared to bone defects below the critical size because it is difficult to induce proper vascularization in the defect center. Significant progress in this field has been made by using stem cells on bone grafts before implantation—prefabricated bioreactor models that partially already contain new donor bone—with the addition of growth factors to the cell medium [25,26]. This has proven to be suitable when the bone defect is surrounded by old bone on at least three sides, i.e., with intact bone integrity. This is also shown by studies in which bone was reconstructed by filling cylindrical bone defects in rat tibia [27], rabbit calvaria [10], or sheep femoral bone [28]. Due to the complex tissue regeneration in the bone reconstruction of large bone defects, it is often challenging to provide new bone growth in the center of a bone defect. This is due to the lack of vascularization in the center of the defect, and the corresponding impaired oxygen delivery to the cells implanted in the construct [29]. In a previous pilot study, part of the rabbits’ joints was successfully reconstructed using the 3D printed graft based on nHAP, and its shape and dimensions fully matched the joint defect [18]. The nHAP material that degrades at a similar rate as natural bone was used in the study, allowing the surrounding bone to heal properly without fibrous tissue ingrowth. Prior to the in vivo study, the absence of any adverse effect of the 3D-printed graft was validated on SCAPs in direct contact. Furthermore, it was found to be biocompatible, stimulating cell migration and osteodifferentiation [18]. Detailed histological analysis revealed osteoblast migration inside the 3D bone graft, while the formation of blood vessels enabled new bone development nearby. Additionally, the printed nHAP-based graft provided adequate mechanical support and porosity, allowing for the necessary osteoconductivity. The combination of the graft’s materials, its mechanical properties, and its specific 3D structure seems to provide various physical and chemical signals as the main contribution to cell modulation and timing.

Research is still ongoing to identify materials that could effectively promote the regeneration of both bone and cartilage [30,31]. Our results showed that 12 weeks after the replacement of bone and cartilage with a 3D construct of complex composition, regular cartilaginous tissue covered by newly formed cartilage on one side could be observed, while on the other side contact with bone tissue was visible. Also, young chondrocytes and fibroblasts penetrated the pores of the 3D graft on the articular surfaces of the femur and thus proved the presence of chondrogenesis, the process of cartilage regeneration. Regeneration of cartilage was achieved due to a complex gel based on nHAP enriched with hydroxyethyl cellulose, sodium hyaluronate, and chondroitin sulfate. The relative gene expression analysis of tissue corresponds to histological findings. Foxc1 and *SOX9* were significantly upregulated in the experimental group. *SOX9* gene, a pivotal transcription factor, is expressed in cells from the progenitor stage throughout differentiation, and in permanent chondrocytes. Its higher expression ensures successful chondrogenesis [32]. It is suggested that *SOX9* and *FOXC1* cooperatively control endochondral ossification [33], while *ITM2A*, a cell surface chondrogenesis marker, if overexpressed, could potentially inhibit chondrogenic differentiation of mesenchymal stem cells [34]. In our results, *ITM2A* expression was not significantly upregulated but showed a tendency to be higher expressed in comparison to the control. This balance of investigated genes, regulators of chondrogenesis, implies adequate development of new cartilage at the place of the 3D construct.

Prior to the use of nHAP gel in animal models, a preliminary in vitro study investigated its potential effect on stem cells. The gel affected mitochondrial activity of SCAPs, from 82.01 ± 6.79% as 1% solution to 121.67 ± 13.32% as 0.125% solution, in comparison to the control. Although higher concentrated hydrogel reduced slightly cell activity, it remained in high values. According to ISO 10993-5, percentages of cell viability above 70% are considered non-cytotoxic [19]. On the other hand, the lowest concentration (0.125%) significantly stimulated cell activity (*p* < 0.01) after 24 h of incubation, indicating excellent biocompatibility in direct contact with stem cells. As for further experiments of bioactivity, the material was shown to significantly stimulate osteogenic and chondrogenic differentiation when added in low concentration to the stem cells. Similar results for alkaline phosphatase cell production were observed when nHAP hybrid scaffolds enriched with chondroitin sulfate and hyaluronic acid were seeded with osteoblasts [35].

By achieving successful bone regeneration without using stem cells or growth factors, our findings highlight the potential of using engineered bone substitutes with desirable osteoinductive characteristics, as well as the possibility of cartilage tissue regeneration. While our preliminary results are promising, future studies are needed to confirm these results on a larger sample of laboratory animals and in larger animal models and to analyze all the possible limitations of this approach.

## 5. Conclusions

In conclusion, our study demonstrated the promising potential of 3D-printed bone grafts for the simultaneous regeneration of both bone and cartilage, offering a significant step forward in osteochondral tissue engineering. After 12 weeks of healing, we observed near-complete regeneration of bone tissue, with mature, mineralized bone forming at the periphery of the defect, and active osteogenesis in tissue. Furthermore, the incorporation of nHAP gel enhanced the regenerative capacity by promoting cartilage formation, as evidenced by the presence of newly formed cartilaginous tissue. While the results are encouraging, further research is needed to confirm these findings in larger animal models. Also, the long-term outcomes of this approach should be explored.

## Figures and Tables

**Table 1:** Primers with corresponding sequences used in the study.

*BMP4*	Rv	5′ GGG TGA GTG GAT GG AAC 3′
	Fw	5′ CTC GAT GAG TAT GAT AAG GTG GTA 3′
*RUNX2*	Rv	5′ GTC TCG GTG GCT GGT AGT GA 3′
	Fw	5′ ACA AAC AAC CAC AGA ACC ACA AGT 3′
*ALP*	Rv	5′ ATG GCA GTG AAG GGC TTC TT 3′
	Fw	5′ CCA CGT CTT CAC ATT TGG TG 3′
*ITM2A*	Rv	5′ GTCCTGCCAAGATGAATGAA 3′
	Fw	5′ AGGATCTCCTCTTGCAGTC 3′
*FOXC1*	Rv	5′ CGGGGCTCTCGATCTTGGGCA 3′
	Fw	5′ AAGACCGAGAACGGTACGTG 3′
*SOX9*	Rv	5′ CTC TTT TGC ACC CCT CCC ATT 3′
	Fw	5′ GAC TTC ACA TGT CCC AGC ACT A 3′
*GAPDH*	Rv	5′ CCC TGT TGC TGT AGC CAA ATT CGT 3′
	Fw	5′ TCA TGA CCA CAG TCC ATG CCA TCA 3′

**Table 2:** Stereological parameters of the analysis of cartilage and bone tissue in the region of the lateral femur condyle replaced with the personalized 3D graft. Twelve samples in each group (control and 3D graft) were analyzed (4 samples per animal).

Parameters	Control	3D Graft
Volume density of chondrocytes (mm^0^)	0.362 ± 0.044 *	0.295 ± 0.051
Volume density of cartilage matrix (mm^0^)	0.638 ± 0.076	0.705 ± 0.079
Number of chondrocytes	24,805.1 ± 3007.4 ***	17,545.6 ± 2107.4
Numerical density of chondrocytes (mm^−3^)	3664.4 ± 417.8 **	2931.4 ± 386.2
Surface area of chondrocytes (μm^2^)	238.5 ± 22.6	305.7 ± 28.6 ***
Surface area of chondrocytes’ nuclei (μm^2^)	75.3 ± 9.9	98.6 ± 10.2 ***
Surface area of lacunae of cartilage (μm^2^)	136.5 ± 12.4	199.4 ± 16.5 ****
NCR of chondrocytes	0.323 ± 0.021	0.402 ± 0.033 ***
Volume density of osteocytes (mm^0^)	0.266 ± 0.019 **	0.225 ± 0.017
Volume density of bone matrix (mm^0^)	0.560 ± 0.044 ***	0.447 ± 0.041
Volume density of blood vessels in bone tissue (mm^0^)	0.174 ± 0.015	0.328 ± 0.024 ****
Number of osteocytes	28574.2 ± 2314.6 ****	21759.1 ± 2219.4
Numerical density of osteocytes (mm^−3^)	3015.6 ± 353.3 *	2554.5 ± 311.1
Surface area of osteocytes (μm^2^)	172.6 ± 15.4 ***	139.3 ± 13.2
Surface area of osteocytes nuclei (μm^2^)	69.4 ± 8.4	60.5 ± 8.2
Nucleocytoplasmic ratio (NCR) of osteocytes	0.325 ± 0.031	0.409 ± 0.038 ***
Diameter of blood vessels (µm)	55.5 ± 6.6	68.4 ± 7.2 **

* *p* < 0.05, ** *p* < 0.001, *** *p* < 0.0001, and **** *p* < 0.00001.

**Figure A1:**
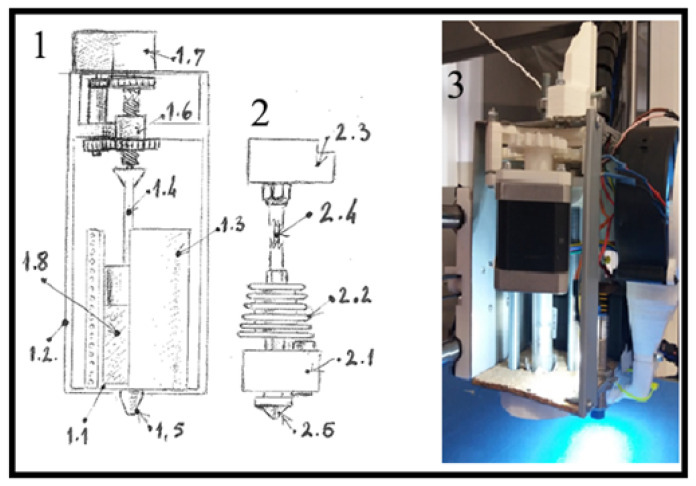
Schematic representation of the extruder design in a custom-made laboratory 3D printer: from technical reasons, the 3D printer was modified to include two extruders: 1—syringe extruder; 1.1—glass syringe; 1.2—external frame of the extruder 1; 1.3—electrical heater coil; 1.4—metal piston of the syringe; 1.5—syringe nozzle (0.8 mm in diameter); 1.6—gears of the piston drive; 1.7—piston drive motor with reduction; 1.8—HAP PLGA mixture; 2—PLGA extruder; 2.1—heater block of the PLGA extruder; 2.2—extruder cooler; 2.3—PLGA extruder motor drive with reduction; 2.4—PLGA filament 1.75 mm in diameter; 2.5—PLGA extruder nozzle 0.4 mm in diameter; 3—paste extruder attached to the head of the printer, with removed electrical heater coil, so the syringe with the metal piston can be visible. (Figure originates from the PhD thesis: Micic M. [2023]. Influence of the choice of protocols on the accuracy of three-dimensional medical models, surgical guides, and bone replacement. School of Medicine, University of Belgrade, National Repository of Dissertations in Serbia, https://nardus.mpn.gov.rs/bitstream/id/155980/Disertacija_14177.pdf. accessed on 1 April 2025).

## Data Availability

The original contributions presented in this study are included in the article. Further inquiries can be directed to the corresponding author.

## Abbreviations

The following abbreviations are used in this manuscript:

3D	three-dimensional
nHAP	nanohydroxyapatite
PLGA	Poly(lactide-co-glycolide)
SCAPs	stem cells from apical papilla
ALBO-OS	name of material based on nHAP and PLGA
BMP2	bone morphogenetic protein 2
PEVA	polyethylene vinyl acetate
PEVV	polyethylene vinyl versatate
SHMP	sodium hexametaphosphate
HEC	hydroxyethyl cellulose
SH	sodium hyaluronate
CH	chondroitin sulfate
CBCT	cone beam computed tomography
STL	Standard Tessellation Language
RKA	randomized Kruskal’s maze-generating algorithm
MTT	3-[4,5-dimethylthiazol-2-yl]-2,5-diphenyl-tetetrazolium bromide
DMSO	dimethyl sulfoxide
RNA	Ribonucleic acid
DNA	Deoxyribonucleic acid
qPCR	quantitative polymerase chain reaction
BMP4	bone morphogenic protein 4
RUNX2	runt-related transcription factor 2
ITM2A	integral membrane protein 2A
FOXC1	forkhead box C1
SOX9	SRY-box transcription factor 9
GAPDH	Gene glyceraldehyde-3-phosphate dehydrogenase
NCR	Nucleocytoplasmic ratio
ALP	alkaline phosphatase

## Appendix A

### Custom 3D Printer Fabrication

To create personalized bone constructs for planned rabbit femur defects, a custom 3D printer was developed at the Center of Bone Biology based on the Prusa I3 fused deposition modeling (FDM) printer (Prusa Research, Praha, Czech Republic). Key modifications included replacing the original frame with a robust, welded single-piece aluminum cube and upgrading the axis rails from 8 mm to 20 mm for added stability. A paste extruder for nHAP, designed as a syringe extruder with a NEMA 17 motor (6:1 gear ratio) and a 10cc steel–glass syringe, was added alongside a thermoplastic extruder and a 45-vat power air turbine on the X-axis. These upgrades allowed the printer to carry both extruders and the air turbine, minimizing vibrations during printing.
